# Nanopipette/Nanorod-Combined Quartz Tuning Fork–Atomic Force Microscope

**DOI:** 10.3390/s19081794

**Published:** 2019-04-15

**Authors:** Sangmin An, Wonho Jhe

**Affiliations:** Department of Physics & Astronomy, Seoul National University, Seoul 08826, Korea

**Keywords:** nanopipette, nanorod, QTF–AFM, nanolithography, nanoscratching

## Abstract

We introduce a nanopipette/quartz tuning fork (QTF)–atomic force microscope (AFM) for nanolithography and a nanorod/QTF–AFM for nanoscratching with in situ detection of shear dynamics during performance. Capillary-condensed nanoscale water meniscus-mediated and electric field-assisted small-volume liquid ejection and nanolithography in ambient conditions are performed at a low bias voltage (~10 V) via a nanopipette/QTF–AFM. We produce and analyze Au nanoparticle-aggregated nanowire by using nanomeniscus-based particle stacking via a nanopipette/QTF–AFM. In addition, we perform a nanoscratching technique using in situ detection of the mechanical interactions of shear dynamics via a nanorod/QTF–AFM with force sensor capability and high sensitivity.

## 1. Introduction

A nanopipette/nanorod is a tool for the control of micro/nanoscale objects and the investigation of their intrinsic properties in nanoscale science and technology, especially nanobiology [1,2]. Generally, they are used for biological or medical applications in the liquid/soft environment, such as biomolecule cell injection [3], manipulation of micro/nanoscale biomolecules [4], and nanobioengineering [5]. Moreover, they are cheap and easily fabricated by using a mechanical puller for fresh usage and the delivery of a small amount of liquid or the precise manipulation of micro/nanoobjects in the soft matter environment. However, in the case of hard surfaces, the tip of the nanopipette/nanorod apex is easily broken by approaching the surface without the guidance of a precise distance-controlling apparatus for the purpose of nanolithography, such as an atomic force microscope (AFM). Several research studies related to nanolithography have used a conventional AFM system, such as dip-pen lithography [6] corresponding to the proposed nanopipette/quartz tuning fork (QTF)–atomic force microscope (AFM) and cantilever-based nanoscratching [7,8] corresponding to the proposed nanorod/QTF–AFM. However, it is expensive to fabricate the dip-pen cantilever to deliver a small amount of liquid for performing dip-pen lithography, and it is difficult to avoid the wear of the tip apex without expensive diamond coating treatment on the cantilever apex. In addition, the conventional cantilever of an AFM cannot handle the relatively massive tip of the nanopipette and nanorod in the case of direct attachment onto the cantilever body due to geometrical issues (low stiffness under 100 N/m, tapping mode oscillation). Here, we describe the precise control of a nanopipette/nanorod combined with a quartz tuning fork (QTF)-based AFM system with shear mode/small oscillation operation and fresh usage without tip breaking. The relatively massive tip of the nanopipette/nanorod is clearly attached on one prong of the QTF, which shows the least degradation of the quality (*Q*-) factor due to the high stiffness of the QTF itself (~27,000 N/m), while the tip apex detects lateral interactions during nanolithography and nanoscratching. The system has the capability to perform versatile experiments (nanolithography, nanoscratching, and sensor) with the same QTF–AFM system by only exchanging the nanopipette and nanorod tip. Nevertheless, there is a critical drawback of the system, which is that the QTF sensor has temperature and humidity dependencies. Thus, we need to control the temperature and humidity tightly as constants while managing experimental conditions.

First, we demonstrate a nanopipette/QTF–AFM for small liquid delivery-based nanolithography with in situ detection of shear interactions during performance. We performed an electric field-assisted small-volume liquid ejection in ambient conditions at a bias voltage (10 V) via a non-contact, distance-controlled-within-10-nm nanopipette/QTF–AFM; the systematic study of the system is described in previous work [9]. The pencil-shaped nanopipette is itself a versatile item for research on biological topics, such as sensors [10], scanning ion conductance microscopy (SICM) [11], manipulation of nano-objects [12], and delivery control [13]. However, most such studies have been performed in liquid environments, and thus, they have not addressed the challenges of direct ejection through the nanoscale aperture of the nanopipette in ambient conditions such as low-volume dispersion on a hard substrate. This is because the apex of the nanopipette is fragile and easily broken when it contacts the hard surface without precise distance control between the tip and surface for the purpose of nanolithography. We demonstrate nanolithography via a nanopipette/QTF–AFM with the realization of nanoscale water meniscus-mediated electric field-assisted liquid ejection through the nanoscale aperture of the nanopipette down to 30 nm in ambient conditions, which provides non-contact, precise distance control (within 10 nm) with no percussion of the nanopipette’s apex.

With the same apparatus, we produce and analyze Au nanoparticle-aggregated nanowire using a nanofabrication technique involving nanomeniscus-based Au nanoparticle stacking via a nanopipette/QTF–AFM by changing the tip movement direction from lateral to vertical. We demonstrate nanofabrication via a nanopipette/QTF–AFM with direct non-template fabrication and analyze Au nanoparticle-aggregated nanowire using nanomeniscus-based particle stacking in ambient conditions. Nanofabrication is essential for the realization of molecular architecture and nanobioengineering in nanoscience and technology. Several nanofabrication methods have been proposed, such as beam-based writing [14,15] and pen-type lithography [6,16] categorized as top-down and bottom-up fabrication, respectively. Among the nanofabrication methods, we choose pen-type nanofabrication with an Au nanoparticle solution with vertical retraction of the tip instead of lateral drawing, which has substantial advantages. These include the following: (i) 3-D nanofabrication of nanowires, (ii) electric-field free (resolving usage of particular ink and substrate) nanofabrication, (iii) non-template fabrication and in situ analysis of the mechanical properties of fabricated nanowires, and (iv) avoidance of tip wear with precise AFM control.

Then we perform nanoscratching with in situ detection of the mechanical shear interactions via a buckled nanorod tip-combined QTF–AFM. Instead of the nanopipette, we demonstrate a nanoscratching technique involving the nanorod tip (pulled quartz rod) with a QTF–AFM with in situ sensing of the mechanical interactions with a high *Q*-factor (4000–8000) in ambient conditions. We show several advantages compared with the conventional contact-mode AFM-based nanoscratching technique for nanotechnology applications [7,8,17,18] including investigations of intrinsic properties (frictional phenomena) [19,20,21] providing only the static force with wear issue due to direct contact on the surface. These include the following: (i) Varying the scratched linewidth by controlling the pushing depth of the buckled tip with the guidance of the QTF–AFM, (ii) in situ sensing of mechanical interactions, (iii) minimization of tip wear due to the indirect contact of the tip apex with the buckling scheme allowing reliability, and (iv) relatively high stiffness of the quartz tip, which provides reliable usage while maintaining the *Q*-factor for sensing shear dynamics in ambient conditions. Finally, we note the sensor capability of the buckled tip of the nanorod/QTF–AFM in ambient conditions, which was previously investigated [22].

## 2. Materials and Methods

**Figure 1:**
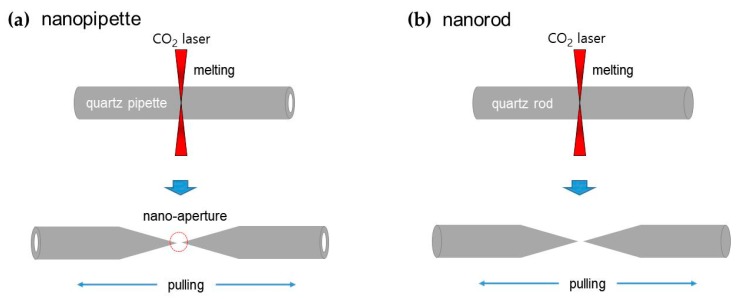
Fabrication of nanopipette (**a**) and nanorod (**b**) by using a mechanical puller (P-2000, Sutter Instruments). After the CO_2_ laser focused on the middle of the quartz pipette (1 mm outer diameter, 0.5–0.7 mm inner diameter) or quartz rod (1 mm diameter) for melting, the machine fabricated two identical pencil-shaped nanopipettes and nanorods by pulling and breaking off the quartz pipette and quartz rod.

We used the pulled nanopipette and nanorod for performing nanolithography and nanoscratching with sensor capability, which were produced by using a mechanical puller (P-2000, Sutter Instruments, Novato, CA, USA). We prepared a quartz pipette (1 mm outer diameter, 0.5–0.7 mm inner diameter) or a quartz rod (1 mm diameter), and the high-energy CO_2_ laser focused on the middle for melting. Then the machine fabricated two identical pencil-shaped nanopipettes (Figure 1a) and nanorods (Figure 1b) by pulling and breaking off the quartz pipette and quartz rod (Figure 1). After the mechanical puller gripped both edges of the 1 mm diameter quartz rod, the high-energy CO_2_ laser focused on the middle of the quartz rod for melting. Finally, pulling started, and it finished with both sharp edges of the pencil-shaped tip with an apex diameter of nanometer scale. Normally, one can choose quartz or borosilicate as materials of a 1 mm rod for fabrication of a pulled nanopipette or nanorod. We chose the quartz due to a high Young’s modulus for reliable performance. The pencil-shaped tip’s tapered angle was about 2 degrees near the tip apex, and thus, the tip instantly buckled on contact and further pushing. We expected that the tapered angle variation and material would influence the sensitivity changes of the system, such as sensitivity maximizing with the optimal tapered angle. The apex diameters were varied from tens of nanometers to micrometers by varying the puller’s control parameters.

**Figure 2:**
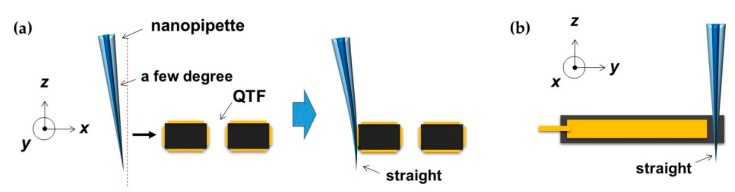
Attachment of the pulled nanopipette, which can be substituted with a nanorod to one prong of the quartz tuning fork (QTF) sensor: (**a**) The tip, inclined a few degrees, is accurately attached on the prong of the QTF perpendicular to the *x*-axis by pushing the tip further after contact with the prong of QTF for accurate force sensing from the apex of the tip; (**b**) installed tip straight to the *y*-axis.

After we fabricated the pulled nanopipette and nanorod, we attached it on one prong of the QTF sensor by manipulating the nanopipette/nanorod holder, which provides mechanical information from the surface. The QTF sensor has two quartz crystal prongs (dimensions: thickness (350 μm), width (600 μm), length (5000 μm)) covered by two isolated electrodes for driving its resonance frequency, which are normally used in analog watches for accurate time measurement. When we drive the QTF with the resonance frequency through the electrodes by using a function generator, the oscillation amplitude of the piezoelectric quartz body produces a maximized value with a high *Q*-factor derived from a current signal from the electrodes. Figure 2 shows the example of the attachment of the pulled nanopipette on one side of the QTF prong, which can be substituted with a nanorod. Note that we used glue-free attachment with a lateral loading force, as depicted in Figure 2, which gives enough stability even if the interaction force (normally less than 1 μN during experiments) affects the apex of the nanopipette. These issues are discussed in a previous study comparing glue and glue-free attachment of nanopipette/QTF–AFM systems [9]. We used an objective lens and a CCD camera to make a precise tip and QTF alignment in *x*-, *y*-, and *z*-axes. We installed the tip onto a translator to control the position of the tip apex on the prong perpendicular to the *y*-axis (normal to the substrate) for straight movement in the *x*-axis and to ensure its stable contact with the QTF prong with its initial small angle in the *x*-axis. Then, the tip was attached on the QTF perpendicular to the *x*-axis by pushing after contact and further pushing for accurate force sensing from the apex of the tip (Figure 2a), and the tip was also installed straight to the *y*-axis (Figure 2b). That is, the initially inclined tip along the *x*-axis was adjusted to be vertical to the substrate after a further push by the rigid QTF prong, which has a high stiffness of about 27,000 N/m, and thus, solid and stable contact was realized between the tip and the QTF.

The temperature and humidity significantly influence experimental results. Thus, we built a double-chamber setup (acrylic outer chamber, metal inner chamber) to sustain temperature (22 ± 0.3 °C) and relative humidity (40.5 ± 0.5%). We obtained a thermal drift value of about 0.4 pm/s while maintaining constant temperature. During nanolithography with a nanopipette, the humidity crucially affects the possibility of patterning and even the pattern width. At low relative humidity, the inside liquid of the nanopipette cannot be extruded, as the capillary-condensed water meniscus is not formed between the tip apex and the substrate. We also used an anti-vibration system on the outside and inside chambers with two commercial floating optical tables to minimize the vibration noise.

## 3. Results

Nanolithography, nanofabrication, nanoscratching, and sensing capability were demonstrated via a nanopipette/nanorod QTF–AFM with precise control of the tip apex through the guidance of the AFM. At the same time, we obtained in situ sensing of mechanical properties (shear dynamics) with the QTF sensor’s recorded amplitude (A) and phase (θ) signals for amplitude modulation QTF–AFM and frequency shift (Δ*F*) and damping coefficient (*g*’) signals for frequency modulation QTF–AFM. The mechanical interactions of the tip were experimentally derived by applying the theory of amplitude and frequency modulation (FM) to the QTF–AFM [23,24,25,26]. We used a simple harmonic oscillator model with external interaction forces:

(1) $$m\ddot{x}+b\dot{x}+kx=F_{0}e^{i\omega_{0}t}+F_{int},$$

where *k* (= 27,439.2 N/m) is the stiffness obtained by calculating the thermal spectrum, $b\;(=k/\left(Q\omega_{0}\right))$ is the damping coefficient in air, $m\;\left(=k/\omega_{0}^{2}\right)$ is the effective mass, *Q* is the quality factor (typically 4000–8000 in ambient conditions), $\omega_{0}\;\left(=2\pi f_{0}\right)$ is the resonance frequency, $F_{0}\;\left(=kA_{0}/Q\right)$ is the amplitude of the drive, $A_{0}$ is the oscillation amplitude in air measured by monitoring its output current signal, and $F_{int}\;\left(=-b_{int}\dot{x}-k_{int}x\right)$ is the nonlinear interaction force. We can obtain the effective elasticity (*k*_int_) and damping coefficient (*b*_int_), elastic force (*F*_k_), viscous force (*F*_b_), and energy dissipation (*E*_dis_) by calculating the detected amplitude and phase signals of the QTF sensor in the case of amplitude-mode QTF–AFM for nanolithography, nanofabrication, and nanoscratching. We can obtain the frequency shift and damping coefficient in frequency-mode QTF–AFM for sensing capability by inserting $x\left(t\right)=x_{0}e^{i\left(\omega_{0}t-\pi /2\right)}$ into Equation (1).

### 3.1. Nanolithography via Nanopipette/QTF–AFM

**Figure 3:**
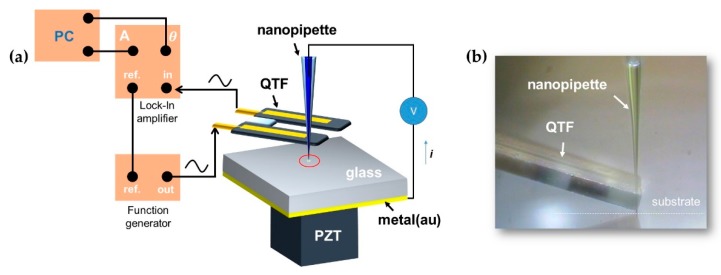
Nanopipette/quartz tuning fork–atomic force microscope (QTF–AFM): (**a**) Schematic of experiment. The nanopipette is attached laterally on the prong of the QTF, and it approaches the surface with the guidance of the QTF–AFM, while the mechanical interactions are recorded by the detection of the amplitude and phase signals of the QTF sensor with a lock-in amplifier and function generator; (**b**) side view of the liquid-filled nanopipette attached on the prong of the QTF sensor.

We demonstrate nanoscale liquid delivery-based nanolithography with in situ detection of shear interactions via a nanopipette/QTF–AFM. Figure 3a shows the schematic of the experiment. After we filled the pulled nanopipettes of various aperture sizes (30 nm to 2 μm) with the Au nanoparticle solution, the tip was attached on the prong of the QTF sensor, and it approached the surface with the precise guidance of the QTF–AFM. The substrate was prepared with glass (thickness: 150 μm) coated with an Au electrode (thickness: 20 nm) on the bottom of the glass surface to prevent the continuous ejection of liquid that occurs with the direct connection of liquid with the surface electrode due to the field-assisted spreading phenomenon of liquid solution on the interest surface. We monitored the mechanical responses by the detection of amplitude and phase signals for the interpretation of elastic and viscous behaviors with a function generator (33120A, Agilent Technologies, Inc., Santa Clara, CA, USA) and a lock-in amplifier (SR830, Stanford Research Systems, Sunnyvale, CA, USA) for driving the QTF sensor’s resonance frequency with a high *Q*-factor in ambient conditions. When we normally drive the QTF sensor with a microscale voltage by a function generator, we sense a current signal of about 0.5 nA passed through a homemade current–voltage converter, which has a 10^7^ gain (10 MΩ) from the QTF electrodes. We obtained a clean current signal from noise cancelation by the lock-in amplifier, which showed a signal-to-noise level of about 80 dB. Figure 3b shows the side view of the liquid-filled nanopipette with the combination of QTF–AFM.

**Figure 4:**
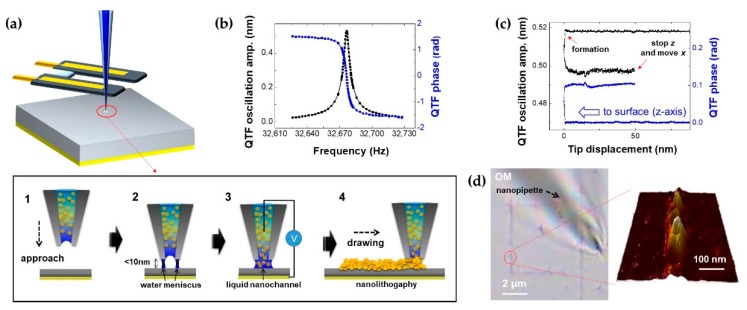
Nanolithography via nanopipette/QTF–AFM. (**a**) Schematic and procedure of experiment: (1) Tip approaching, (2) formation of nanoscale water meniscus between the rim of the tip apex and the substrate, (3) nanomeniscus-based ejection of the Au nanoparticle solution at a voltage bias (~10 V) in ambient conditions forming liquid nanochannel, (4) nanolithography by lateral movement of the tip as drawing. (**b**) Resonance curve of the QTF sensor with high Q-factor (~6000). (**c**) Approach/retraction curve for experimental procedure. (**d**) Nanolithography result (optical microscope (OM) image and AFM scanned image) with less than 100 nm linewidth with 30 nm aperture nanopipette.

Figure 4a shows the schematic of the nanolithography experiment. After we prepared the filled nanopipette and attached it on the prong of the QTF, the tip approached the glass substrate that was coated underneath with the Au thin film (Figure 4a-1). Within 10 nm between the tip apex and the surface, a capillary-condensed nanoscale water meniscus was formed between the rim of the tip apex and the substrate by liquid–vapor phase transition due to the lowered energy barrier [27] (Figure 4a-2). Immediately thereafter, the liquid nanochannel was formed with the guidance of the electric field dominantly exerting near the apex area (Figure 4a-3). Finally, the nanopipette drew the nanoscale pattern (Figure 4a-4). For precise distance control, we used the QTF–AFM system with the driving frequency of the intrinsic resonance of the QTF sensor (32,673.5 Hz) and its dithering amplitude of 0.5 nm, and the measured *Q*-factor was about 6000 (Figure 4b). The graph was obtained after the nanopipette was attached on the one prong of the QTF. Note that the dithering amplitude indicates the oscillation at the edge of the QTF prong, which is derived from the equipartition theorem with thermal noise measurement, and the oscillation amplitude of the nanopipette apex can be calculated with geometrical consideration based on the coupled harmonic oscillator model. In addition, we electrically drove the QTF oscillator, which gave a stray capacitance effect on the resonance curve shape due to the electrodes located on the QTF body. We removed the stray capacitance effect with a parametric capacitor compensation scheme, which allowed the Lorentzian shape of the resonance curve for accurate quantitative measurement. Figure 4c shows the approach/retraction curve for the formation of the liquid nanochannel. When the tip approached within 10 nm, the nanoscale water bridge was suddenly formed. Then, the tip retracted immediately, stopped at about a 50 nm distance from the formation spot, and started to move laterally for nanolithography. Note that one can produce a continuous straight-line shape with this drawing scheme, as there is sufficient space of 50 nm when the tip is clearly aligned perpendicular to the surface with the angle tilt stage. However, instead of this, one can also achieve a straight line with non-contact feedback control by maintaining the distance when the tip is not clearly aligned. Figure 4d shows the optical microscope (OM) image and AFM scanned image of the directly drawn nanolithography result with less than 100 nm linewidth through a 30 nm aperture nanopipette with the filled Au nanoparticle solution.

### 3.2. Nanofabrication via Nanopipette/QTF–AFM

**Figure 5:**
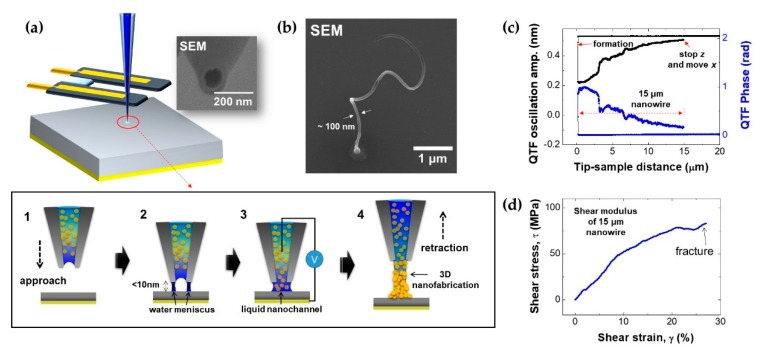
Nanofabrication via nanopipette/QTF–AFM. (**a**) Schematic and procedure of experiment. When the nanopipette tip approached the substrate (1), the nanoscale water meniscus was formed between the rim of the tip apex and the substrate (2) and nanomeniscus-based ejection of the 2 nm Au particle solution occurred at a voltage bias of ~10 V in ambient conditions with formation of the liquid nanochannel (3). Then, the Au nanoparticle-aggregated nanowire formed as the tip retracted in 3D space while the liquid solution evaporated (4). (**b**) Scanning electron microscope (SEM) image of the 100 nm diameter fabricated Au nanoparticle-aggregated nanowire. (**c**) Approach/retraction curve of the nanowire fabrication process. (**d**) Shear stress measurement of the fabricated nanowire with a nanopipette/QTF–AFM.

We show a nanofabrication technique for producing and analyzing an Au nanoparticle-aggregated nanowire via nanomeniscus-assisted nanoparticle stacking by using a nanopipette/QTF–AFM by changing the tip movement direction from lateral to vertical. Figure 5a shows the fabrication procedure schematic of the vertically grown Au nanoparticle-aggregated nanowire. Glass (thickness: 150 μm) was used for the substrate, and Au electrode coating (thickness: 20 nm) was coated on its bottom surface to prevent the electric field-assisted spreading of liquid on the interest surface. Afterwards, we filled a 100 nm aperture nanopipette (scanning electron microscope (SEM) image in Figure 5a) with a 2 nm Au nanoparticle solution, and performed the nanofabrication of vertically grown Au nanoparticle-aggregated nanowire. The fabrication process of the vertical growth is shown in the magnified spot of interest. After the tip apex approached the substrate within 10 nm (Figure 5a-1), the nanoscale water meniscus [15] formed between the rim of the tip apex and the surface (Figure 5a-2). Moreover, the inside liquid was ejected onto the substrate by the attractive force, which formed the liquid nanochannel (Figure 5a-3). After that, the tip retracted immediately to the opposite direction (*z*-axis), while the solution continued to eject and formed a particle-stacked and aggregated nanowire while the liquid evaporated (Figure 5a-4). Figure 5b shows SEM images of the Au nanoparticle-aggregated nanowire with a uniform diameter (100 nm) and length of ~5 μm. Figure 5c shows the amplitude and phase signals of the QTF sensor for the definition of the length of the fabricated nanoparticle-aggregated nanowire, which was easily and precisely controlled by the vertical retraction distance from the glass substrate with the guidance of the piezoelectric transducer of the AFM. When the tip approached the surface within 10 nm, the amplitude drastically decreased, indicating the formation of the liquid nanochannel. Then, we retracted the tip while monitoring and gradually increasing the amplitude signal, which decreased the nanowire’s stiffness by increasing the length until it reached the required length (e.g., 15 μm). Next, we moved the plate laterally to sense the mechanical responses of the nanoparticle-aggregated nanowire until the nanowire was fractured at 80 MPa of shear stress, which was derived from *τ = F_s_/A* (*τ*: shear stress, *F_s_*: shear force by integration of the measured force gradient, *A*: circular cross-section area of the nanowire) (Figure 5d).

### 3.3. Nanoscratching via Nanorod/QTF–AFM

**Figure 6:**
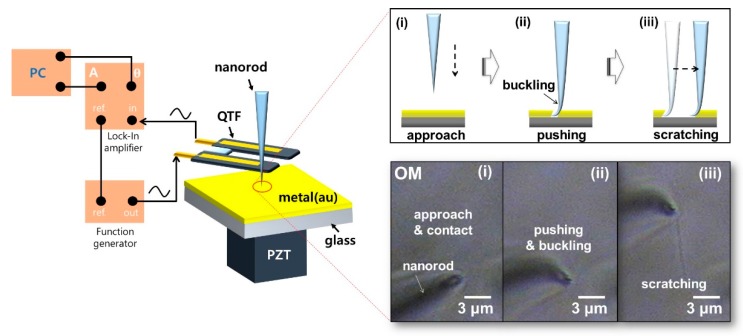
Nanoscratching via nanorod/QTF–AFM. The experimental procedure was as follows: (i) contact, (ii) buckling, and (iii) nanoscratching. After the buckled tip was ready by pushing, we started the lateral motion to perform nanoscratching. The OM located under the interest substrate was used to observe nanoscratching phenomena.

We perform a nanoscratching technique via a nanorod tip with a QTF–AFM instead of a nanopipette with in situ detection of the mechanical shear interactions. We chose the pulled quartz nanorod tip for nanoscratching (which avoids the contact percussion problem due to the high Young’s modulus of the quartz tip and apex) instead of the nanopipette tip, because the apex of nanopipette tip has a fragile apex, as the hollow shape is easily broken by further pushing on the surface. Note that the buckled nanorod tip minimizes the tip wear allowing sidewall contact, which dominantly contributes to nanoscratching, with avoidance of direct contact of the tip apex resolving the wear issue of the tip apex. To increase the safety of the tip apex, we initially tilted the angle of the tip a few degrees and approached the tip on the surface. Thus, the tip immediately started to buckle when the tip contacted the surface without any breaking of the apex. Figure 6 shows the experimental system and nanoscratching OM images. We prepared the nanorod by pulling the quartz rod (diameter: 1 mm), and we attached it on the prong of the QTF, which gave mechanical information on the substrate coated with Au (thickness: 40 nm). After the tip was attached on the QTF sensor, the experimental process was as follows: (i) contact by approach, (ii) buckling by pushing, (iii) scratching with lateral motion. We watched the surface nanoscratching phenomena by using an OM located under the interest substrate [28].

**Figure 7:**
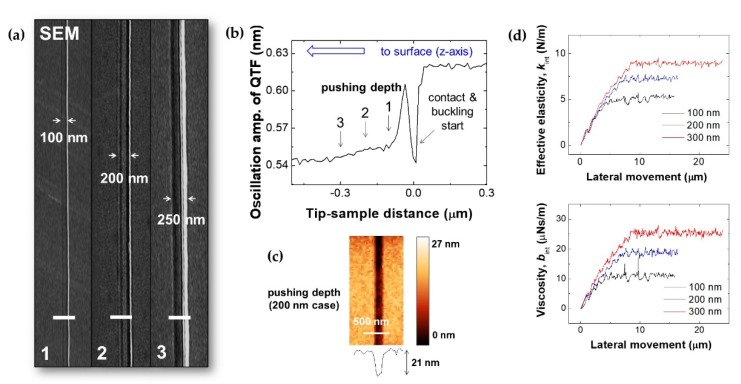
Nanoscratching via nanorod/QTF–AFM: (**a**) SEM images of the scratched linewidths with ~100 nm (1), ~200 nm (2), and ~250 nm (3); (**b**) amplitude approach curve of QTF sensor (*z*-axis) for definition of pushing depth; (**c**) AFM images and depth profiles (trench depth: 25 nm) for pushing depth of 200 nm; (**d**) detected *k*_int_ and *b*_int_ by lateral motion according to pushing depth (100–300 nm).

We studied pushing depth (normal load) dependence on Au-coated glass by the nanoscratching technique with sensing of mechanical properties (viscoelasticity) through the QTF sensor’s amplitude and phase signals. Figure 7a shows SEM images of straight nanoscratched lines (1–3) according to pushing depth. Depending on the pushing depth from 100 nm to 300 nm, defined by the QTF–AFM, we varied the scratched linewidths from 100 nm to 250 nm while we observed the scratching phenomena using an OM under the interest surface for accurate tip positioning. Figure 7b shows the approach amplitude curve of the QTF, which indicates the pushing depth. When the tip contacted the surface, the amplitude drastically decreased and increased by further pushing due to tip buckling, and then the amplitude decreased gradually (1–3 in Figure 7b). Figure 7c shows AFM images with depth profiles of nanoscratched lines (trench depth: 25 nm) in the case of 200 nm pushing depth. Figure 7d shows the detected *k*_int_ and *b*_int_ according to pushing depth (1–3 in Figure 7d, 100–300 nm) with lateral motion, which were obtained from calculating the QTF sensor’s amplitude and phase signals with the damped harmonic oscillator model. After we parked the tip at each pushing depth (100–300 nm), we moved the tip laterally with the lateral movement of the piezoelectric transducer and sensed the shear dynamics. As a result, the detected *k*_int_ and *b*_int_ increased by lateral motion until it reached the saturation point, and then the tip started to move laterally by overcoming the static friction and show saturation behavior. We observed increasing behavior of the detected saturation values of *k*_int_ and *b*_int_ by increasing the pushing depth, as *k*_int_ ≈ 5.1, 7.3, and 9.0 N/m and *b*_int_ ≈ 12.0, 18.8, and 25.5 μNs/m for pushing depths of 100, 200, and 300 nm, respectively.

### 3.4. Buckled Tip-Based Sensitive Sensor via Nanorod/QTF–AFM

**Figure 8:**
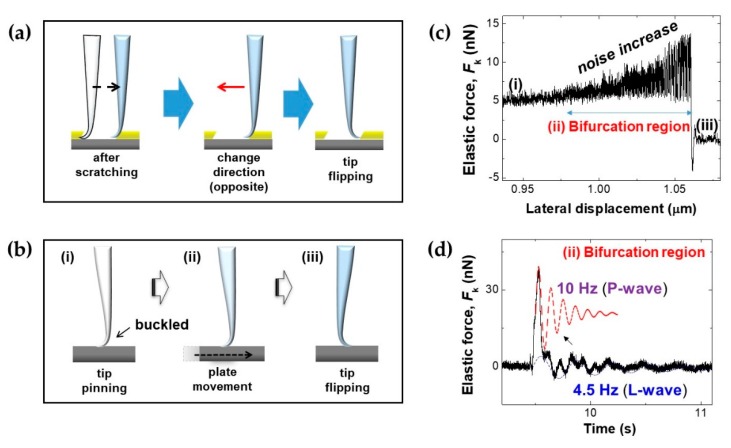
Buckled tip-based mechanical sensor. (**a**) Reversing the buckled tip in the opposite direction for additional nanoscratching. (**b**) Experimental procedure: (i) approaching and buckling the nanorod tip, (ii) plate movement in the lateral direction, (iii) abrupt flipping of the buckled tip. (**c**) Noise increase with increase of sensitivity in the region of bifurcation (data from [22]. (**d**) Sensing of mechanical perturbations transferred from the impact on the optical table (data from [22]).

We note the sensor capability of the buckled tip of the nanorod/QTF–AFM in ambient conditions, which was previously investigated as a bifurcation-enhanced sensitive detection technique [22]. After we performed nanoscratching with a nanorod/QTF–AFM, we reversed the buckled tip in the opposite scratching direction for additional nanoscratching (Figure 8a). Right before the flipping of the buckled tip was executed, we found the sensitivity increased when the buckled tip stopped in the bifurcation region. Thus, we precisely investigated this phenomenon with only the glass substrate with a nanorod/QTF–AFM (Figure 8b). The experimental process was as follows: (i) tip contact by approach with guidance of the QTF–AFM and buckling by gently pushing, (ii) lateral plate movement for tip experiencing softening with increase of noise in bifurcation region, resulting in increased sensitivity, (iii) tip flipping. Figure 8c shows evidence of the sensitivity increase with the noise increase during its lateral movement: *F*_k_, derived from the raw data of Δ*F*, and *g*’ via the FM QTF–AFM detection scheme [10]. *F*_k_ increased gradually due to an increase of the lateral force on the surface until the tip flipped. We observed a noise increase in the bifurcation region right before flipping attributed to the mechanical instability of the buckled tip. Figure 8d shows a demonstration of a sensitive mechanical sensor with sensitive measurement of the perpendicular wave (P-wave) and lateral wave (L-wave). We parked the tip in the bifurcation region (noise-increased area in Figure 8c) to sense the mechanical interactions transferred from the impact spot on the table, where we dropped a coin (mass: 5.5 g) from the height of 25 cm. The gravitational potential energy of the coin (25 cm position) was about 1.348 × 10^−2^ N·m transferred through the body of the AFM system facilitated on a vibration isolator. After looking more closely at the measured *F*_k_, we observed two damped oscillatory motions of ~10 Hz (perpendicular wave) and ~4.5 Hz (lateral wave), which were perpendicular and lateral oscillations of the experimental chamber caused by the impact of the coin.

## 4. Conclusions

In conclusion, we have introduced nanolithography and nanofabrication of Au nanoparticle-aggregated nanowire via a nanopipette/QTF–AFM and nanoscratching and highly sensitive detection of mechanical responses as a force sensor via a nanorod/QTF–AFM. With the nanopipette technique, small amounts of diverse nanomaterial solutions can be ejected, and nanolithography and nanofabrication can be performed on substrates in ambient conditions to investigate the biological processes associated with organic/inorganic molecules’ behaviors. With this nanorod technology, nanoscratching with viscoelastic behaviors in wear physics, including further improvement of the convergence technique, can be studied. In addition, an extremely sensitive detection system can be developed for the repeated sensing of the weak surface acoustic waves and the improvement of the mechanical sensor of the nanorod/QTF–AFM.
